# Linac-based stereotactic radiosurgery and fractionated stereotactic radiotherapy for vestibular schwannomas: comparative observations of 139 patients treated at a single institution

**DOI:** 10.1093/jrr/rrt121

**Published:** 2013-10-18

**Authors:** Putipun Puataweepong, Mantana Dhanachai, Somjai Dangprasert, Ladawan Narkwong, Chomporn Sitathanee, Thiti Sawangsilpa, Taweesak Janwityanujit, Pornpan Yongvithisatid

**Affiliations:** 1 Radiation and Oncology Unit, Department of Radiology, Faculty of Medicine, Mahidol University, Bangkok, 10400, Thailand; 2 Department of Surgery, Faculty of Medicine, Ramathibodi Hospital, Mahidol University, Bangkok, 10400, Thailand; 3 Radiosurgery Center, Faculty of Medicine, Ramathibodi Hospital, Mahidol University, Bangkok, 10400, Thailand

**Keywords:** SRS, SRT, vestibular schwannomas, local control, hearing preservation

## Abstract

Stereotactic radiosurgery (SRS) and fractionated stereotactic radiotherapy (SRT) have been recognized as an alternative to surgery for small to medium sized vestibular schwannoma (VS). This study analysed and compared the outcomes of VS treated with the first Thailand installation of a dedicated Linac-based stereotactic radiation machine using single-fraction radiosurgery (SRS), hypofraction stereotactic radiotherapy (HSRT) and conventional fraction stereotactic radiotherapy (CSRT). From 1997 to 2010, a total of 139 consecutive patients with 146 lesions of VS were treated with X-Knife at Ramathibodi hospital, Bangkok, Thailand. SRS was selected for 39 lesions (in patients with small tumors ≤3 cm and non-serviceable hearing function), whereas HSRT (79 lesions) and CSRT (28 lesions) were given for the remaining lesions that were not suitable for SRS. With a median follow-up time of 61 months (range, 12–143), the 5-year local control rate was 95, 100 and 95% in the SRS, HSRT and CSRT groups, respectively. Hearing preservation was observed after SRS in 75%, after HSRT in 87% and after CSRT in 63% of the patients. Cranial nerve complications were low in all groups. There were no statistically significant differences in local control, hearing preservation or complication between the treatment schedules. In view of our results, it may be preferable to use HSRT over CSRT for patients with serviceable hearing because of the shorter duration of treatment.

## INTRODUCTION

Stereotactic radiosurgery (SRS) and radiotherapy (SRT) are techniques which administer precisely directed, high-dose irradiation that tightly conforms to an intracranial target in order to create a desired radiobiologic response, and to minimize radiation dose to the target's surrounding normal tissues. Initial radiosurgery series, using gamma knife (GK) therapy for vestibular schwannoma (VS), have been reported since 1987. Further development of the Linac-based stereotactic technique has allowed it to become an alternative to the GK system. The various fractionation schemes, starting from single higher dose (14–18 Gy), have higher rates of cranial neuropathy [1–5]. This has led to a treatment modality pursuing a lower dose of single-fraction radiosurgery (12–13 Gy) or fractionated stereotactic radiotherapy. The results of tumor control and complication were compared for the 12–13 Gy SRS and the SRT techniques [6]. Previous data from other institutions have shown a local control rate of 92–100%, a hearing preservation rate of 58–65%, and an acceptable 1–5% rate of treatment-related side-effects after the patients had undergone SRS and SRT [7–10]. Although the use of stereotactic radiation for VS nowadays is accepted, the optimal fractionation schedule is still controversial, and the practicality of using SRS and SRT varies according to the level of experience at individual institutions. Since previous studies have only compared two fractionation schedules (SRS vs HSRT, or SRS vs CSRT), the objective of this report was to analyse and compare the long-term outcomes between the three commonly used stereotactic radiation schedules, including single-fraction radiosurgery (SRS), hypofraction SRT (HSRT) and conventional fraction SRT (CSRT) for VS patients treated at the same institution.

## MATERIALS AND METHODS

In 1997, the first Linac-based stereotactic radiation machine in Thailand was opened at the Radiosurgery Center, Ramathibodi Hospital in Bangkok. In this study, we analysed the long-term outcomes for vestibular schwannoma patients treated at our institution.

### Patients

From December 1997 to January 2010, 139 vestibular schwannoma patients with 146 lesions underwent Linac-based SRS/SRT and were included in this study. All patients were followed up prospectively until death. We retrospectively conducted a chart review of this cohort of patients after approval by our institutional ethics committee. The median follow-up time was 61 months (range, 12–143). Prior to treatment, the optimal plan for management of all patients was discussed and approved by our radiosurgery board. A patient who had a tumor large enough for brain stem compression was selected to undergo a maximum safety surgical resection and postoperative radiation for the residual tumor. For a patient with a small to medium sized tumor, SRS or SRT was usually offered; however, for an elderly patient with a tiny lesion, we recommended an observational policy.

An interview and neurological examination focusing on cranial nerve function was performed on all patients before treatment. Hearing assessment was performed by scoring the ability of patients to use the telephone with the affected ear. If the patients were not able to discriminate words or could not hear at all, they were scored as ‘non-serviceable hearing’. Additionally, all patients were sent for audiograms prior to SRS/SRT treatment. Hearing assessment rated according to the Gardner–Robertson classification was recorded, and serviceable hearing was determined as Class I or Class II. Trigeminal nerve function was assessed via asking the patient about facial pain or numbness according to the CTCAE system. Facial nerve function was assessed and scored using the House–Brackmann facial nerve grading system [11]. Evaluation of tumor control, hearing preservation and cranial nerve complication were recorded accordingly for each treated site.

There were 47 males and 92 females in this study. They had a total of 146 lesions, of which 89 (61%) were treated with partial tumor removal before SRS/SRT, and 57 (39%) received SRS/SRT as the primary treatment; of the 146 lesions, 39 (27%) were treated with SRS, whereas 79 (54%) and 28 (19%) were treated with HSRT and CSRT, respectively. Of the 139 patients, 13 had neurofibromatosis type 2 (NF-2). Patient characteristics of each treatment group are detailed in Table 1. Three patients died from unrelated causes. Two patients were lost to follow-up.

**Table 1.** Baseline characteristics of 139 patients with 146 lesions

Parameters	SRS 39 (27%)	HSRT 79 (54%)	CSRT 28 (19%)
Gender			
Male	6 (18%)	28 (37%)	13 (50%)
Female	31 (82%)	48 (63%)	13 (50%)
Surgery			
Yes	25 (64%)	46 (59%)	18 (64%)
No	14 (36%)	33 (41%)	10 (36%)
Genetic predisposition			
Sporadic	39 (97%)	67 (89%)	19 (75%)
NF-2	2 (3%)	6 (11%)	5 (25%)
Hearing function			
Non-serviceable	35 (90%)	46 (58%)	16 (57%)
Serviceable	4 (10%)	33 (42%)	12 (43%)
Age (year)	47 (16–71)	50 (14–78)	39 (18–74)
Tumor size (cm)	1.6 (0.8–3)	2.5 (1–7)	4 (1.1–5)
Tumor volume (cm^3^)	0.96 (0.08–9.2)	3.9 (0.1–34.2)	9.5 (1.7–27.5)

SRS = stereotactic radiosurgery, HSRT = stereotactic radiotherapy, hypofraction, CSRT = stereotactic radiotherapy, conventional fraction. Median follow-up time = 61 months (12–14.3).

### Radiation technique

The SRS/SRT techniques in this study were performed with the linear accelerator-based system [6 MV dedicated LINAC with fixed circular cone (Varian) with X-Knife planning system version 3 & 4 (Radionics}]. In the SRS technique, the Brown–Robert–Wells (BRW) stereotactic frame was applied with the assistance of a neurosurgeon. This differs from the SRT technique in which the relocatable Gill–Thomas–Cosman (GTC) frame was applied. Individual treatment planning was done in a work-station using an image set from a contrast-enhanced CT scan, of 1.25 mm-slice thickness, with or without gadolinium-enhanced MRI. Target and critical organ contouring was done by radiation oncologists, and a treatment plan was generated by medical physicists. The diameter of circular beams ranged from 5–50 mm. The collimator size that covered at least 90% of the target volume was selected. Multiple isocenters were used in irregularly shaped targets. Arc selection was performed, and was mainly non-coplanar. The target volume ratio (TVR) was usually within a range of 1.3–2.

The selection of patients for SRS or SRT technique was based on pretreatment hearing function and tumor size. Patients who had small tumors (≤3 cm) and non-serviceable hearing were usually selected for SRS treatment. Patients who did not have the aforementioned criteria or were not suitable for SRS were selected for HSRT or CSRT treatment. In SRS technique, the prescribed dose might be 12–13 Gy × 1 fraction for a small lesion (≤3cm) with non-serviceable hearing. HSRT was offered to patients with a larger tumor (>3 cm) regardless of hearing levels. In general, the prescribed dose should be 3 Gy × 10 fractions for large irregular lesions near a critical organ, and 5 Gy × 4 fractions, 6 Gy × 3 fractions or 5 Gy × 5 fractions for smaller lesions away from critical organs. However, various dose fractionations for HSRT were selected based not only on tumor factors, such as tumor size, shape and location, but also on individual physician preference and patient expectation. CSRT with the prescribed dose of 1.8–2 Gy × 25 fractions was preserved in very large tumors near critical organs. Table 2 shows the prescribed dose, single dose equivalent, and EQD2 value for SRS/HSRT dose schedules in our study.

**Table 2.** Prescribed dose, Single dose equivalent and EQD2 for SRS and HSRT dose schedules used in our study

SRS/HSRT schedule used in our study			Single dose equivalent (Gy)	EQD2 (Gy)
Total dose (Gy)	Dose/fraction	No. of fractions		
12	12	1	12	36
13	13	1	13	41.6
18	6	3	12	32.4
20	5	4	11	32
25	5	5	12	40
30	3	10	11	36

SRS = stereotactic radiosurgery, HSRT = stereotactic radiotherapy, hypofraction, EQD2 = equivalent dose at 2 Gy = total dose (d+ α/β)/(2+ α/β); assuming α/β =2.

The patients in the SRS group received a median dose of 12 Gy (range, 12–13) prescribed at the 80% isodose line (range, 80–90) to the tumor margins. The median maximum diameter was 1.6 cm (range, 0.8–3). The median tumor volume was 0.96 cm^3^ (range, 0.08–9.2), and the median number of isocenters was 2 (range, 1–8).

The patients in the HSRT group received various hypofraction schedules (5 Gy × 4–5, 6 Gy × 3, and 3 Gy × 10). The median total dose of 25 Gy (range, 18–30) at the 80% isodose line (range, 80–90) was prescribed. A median number of 5 fractions (range, 3–10) was used in the treatment. The median maximum diameter was 2.5 cm (range, 1–7). The median tumor volume was 3.9 cm^3^ (range, 0.1–34.2). The median number of isocenters was 4 (range, 1–8).

The patients in the CSRT group received a median total dose of 50 Gy (range, 45–50) in 25 fractions (range, 20–25) prescribed at the 90% isodose line. The median maximum diameter was 4 cm (range, 1.1–5). The median tumor volume was 9.5 cm^3^ (range, 1.7–27.5). The median number of isocenters was 4 (1–8).

### Follow-up

All patients were seen at 4–6 weeks after completing treatment for a first follow-up visit, then every 6 months for the next 2–3 years. Annual follow-up was continued thereafter. Patients were interviewed with neurological examination focusing on cranial nerve function in V, VII and VIII during each visit. If the patients had new or progressive facial numbness according to the CTCAE grading or an increased House–Brackmann grade, this was scored as trigeminal or facial neuropathy. Regarding hearing function assessment, for any patients who had pre-treatment serviceable hearing, audiograms were repeated every 6–12 months. However, in patients with non-serviceable hearing before treatment, we only asked about their hearing ability in the treated ear without further audiogram. MRI was done yearly for assessment of local tumor control. Local tumor control was defined as stable (no increase in tumor diameter) or decreased maximum tumor diameter on follow-up MRI imaging. Temporary symptomatic tumor necrosis (central tumor necrosis on MRI with temporary increase in tumor size with or without new or worsened neurological symptoms related to the necrosis) but not requiring additional surgical treatment was defined as local tumor control. Local failure was defined as permanent progressive tumor growth with associated symptoms requiring additional surgical treatment.

### Statistical analyses

Demographic data were summarized and compared with respect to the treatment group. Categorical data were described with frequencies and percentages and compared using the Fisher exact tests. Continuous data were reported with medians and ranges and compared using *t*-tests or Wilcoxon rank-sum tests. Local tumor control (LC) and treatment-related complications were calculated using Kaplan–Meier methods, and the survival curves were compared using the log-rank test. Multivariate analysis was done using the Cox proportional hazard model. All statistical analyses were performed using SPSS software, version 16.0.

## RESULTS

All patients could tolerate the treatment very well. There was no interruption of HSRT or CSRT treatments. Minor acute reactions occurring in this study included headaches, nausea and dizziness.

### Local tumor control

**Fig. 1.**
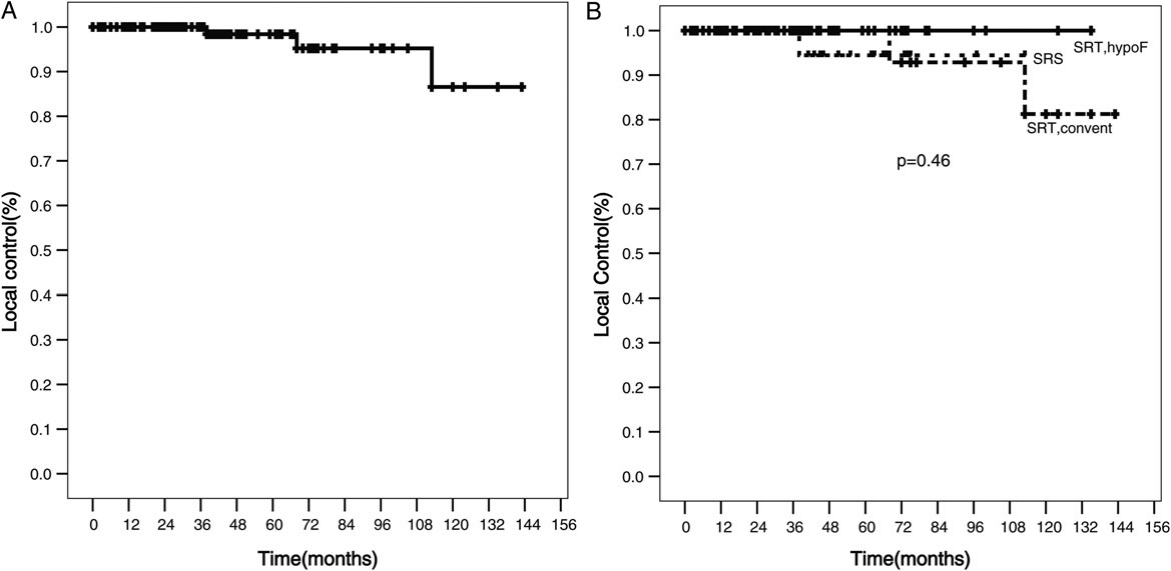
Local control of 146 VS lesions treated with stereotactic radiation, after a median follow-up time of 61 months; local control at 5 and 10 years was 98 and 87%, respectively (**A**). Outcomes were not statistically significantly different after SRS, HSRT or CSRT (*P* = 0.46) (**B**).

The LC rate in all patients was 98% and 87% at 5 and 10 years, respectively. With respect to treatment technique, the 5-year LC rates after SRS, HSRT and CSRT were 95, 100 and 95%, respectively, with no statistically significant difference between the treatments (*P* = 0.46) (Fig. 1). The LC is not statistically significantly different when other factors are compared, including the presence or absence of NF-2, patient age, prior surgical intervention vs no prior surgical intervention, tumor size and tumor volume.

### Hearing preservation

In our study, 49 of 139 patients had serviceable hearing before treatment (33.6%). Three were NF-2 and 46 were sporadic cases. Initially, we selected only patients with small tumors who had non-serviceable hearing for treatment with SRS. However, four patients with serviceable hearing also preferred the SRS and decided to be treated with this technique after being informed about the risks and benefits of all treatment alternatives. The other 33 and 12 patients with serviceable hearing were treated with HSRT and CSRT, respectively. Table 3 shows Gardner–Robertson (GR) classification changes from GR I–II (serviceable hearing) to GR III–V (non-serviceable hearing). The probability of maintaining serviceable hearing did not differ between each radiation technique. With regard to pretreatment GR classification and hearing preservation, 10 out of 13 (76%) GR I patients and 30 out of 36 (83%) GR II patients were able to maintain serviceable hearing function. No GR I patients developed to GR IV, in contrast with four patients in GR II who changed to GR IV hearing function.

**Table 3.** Gardner–Robertson classification changes from GR I–II (serviceable hearing) to GR III–V (non-serviceable hearing) after SRS and SRT

	SRS (*n* = 4)		HSRT (*n* = 33)		CSRT (*n* = 12)	
Before	GR I = 1	GR II = 3	GR I = 2	GR II = 31	GR I = 10	GR II = 2
After	GR I = 1	GR IV = 1	GR III = 1	GR IV = 3	GR III = 2	GR III = 2

GR = Gardner–Robertson, SRS = stereotactic radiosurgery, HSRT = stereotactic radiotherapy, hypofraction, CSRT = stereotactic radiotherapy, conventional fraction.

**Fig. 2.**
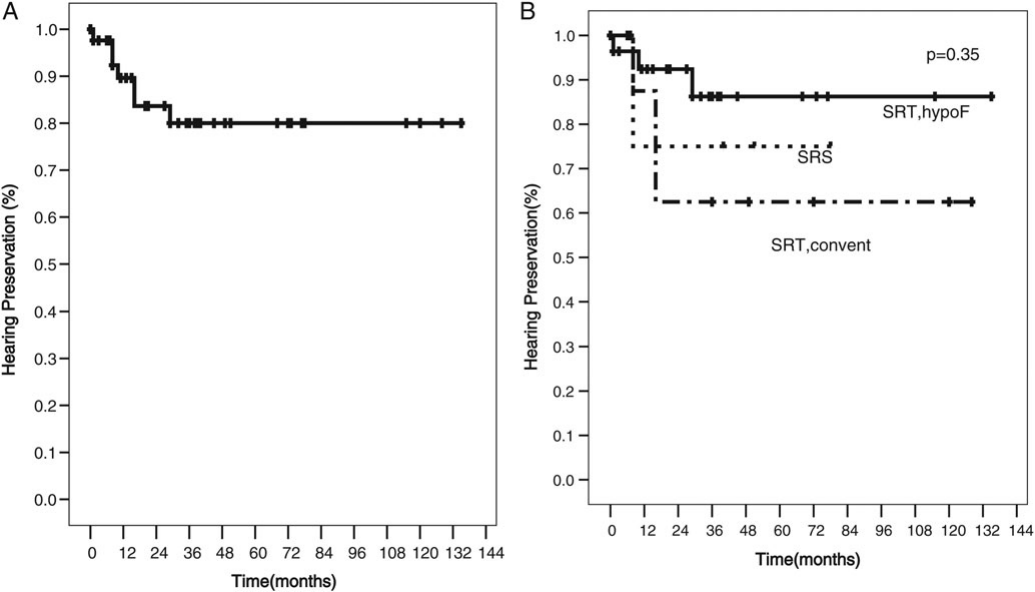
Hearing preservation rate at 1, 2 and 5 years was 90, 84 and 80%, respectively (**A**). Outcomes were not statistically significantly different after SRS, HSRT or CSRT *(P* = 0.35) (**B**).

**Fig. 3.**
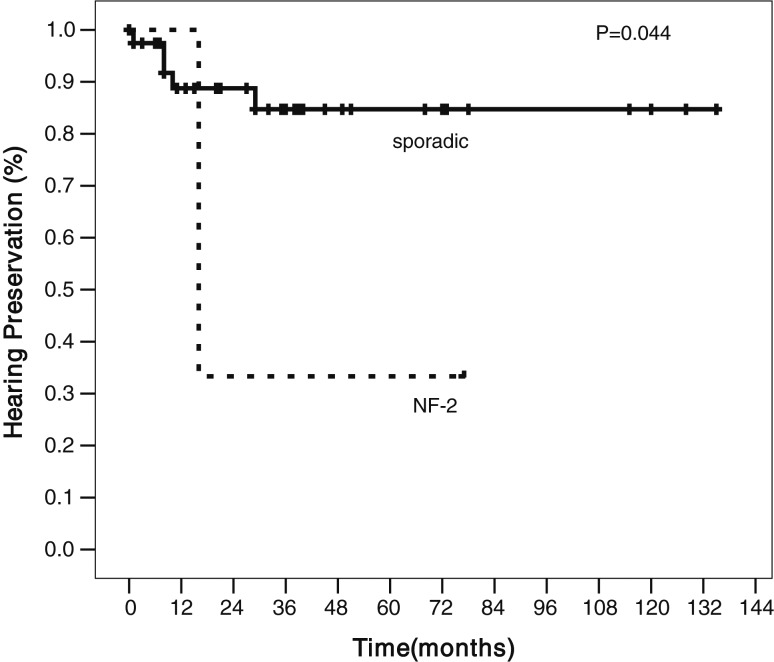
Hearing preservation rate was statistically significantly lower in NF-2 than in sporadic cases (*P* = 0.044).

The overall hearing preservation rates at 1, 2 and 5 years were 90, 84 and 80%, respectively. The 5-year hearing preservation rates after SRS, HSRT and CSRT were 75, 87 and 63%, respectively, with no statistically significant difference (*P* = 0.35) (Fig. 2). The only factor that affected the hearing preservation was the presence of NF-2, and this was statistically significantly lower in NF-2 patients (*P* = 0.044) (Fig. 3)*.* Out of the three NF-2 patients with serviceable hearing, two patients received CSRT, and both of them later developed hearing deterioration. Another patient who received HSRT had maintained hearing function at the time of the last follow-up.

### Trigeminal nerve function

Before SRS/SRT, 32 patients presented with mild to moderate facial numbness. Nine patients (28%) were treated with SRS, while 16 patients (50%) and 7 patients (22%) were treated with HSRT and CSRT, respectively.

After SRS/SRT, 10 of these patients (47%) exhibited improvement of facial numbness (3 in the SRS, 5 in the HSRT and 2 in the CSRT groups). None of these patients showed any worsening of their facial numbness. There was only one patient (0.93%) in the HSRT group who developed new trigeminal neuropathy, characterized by mild facial numbness (CTCAE Grade I). However, the symptom was spontaneously resolved without any specific treatment within 3 months.

### Facial nerve function

Before SRS/SRT, 48 patients presented with facial neuropathy. Of these, 17 patients (35%) were treated with SRS, 21 patients (44%) were treated with HSRT and 10 patients (21%) were treated with CSRT.

After SRS/SRT, five patients (10%) reported completely resolved facial palsy (one in the SRS, two in the HSRT and two in the CSRT groups). Seven patients (15%) reported improvement of their facial palsy. One patient in the SRS group reported worsening of facial palsy (the House–Brackmann score changed from 2 to 3). Three patients (3.2%) developed new facial neuropathy (a House–Brackmann score of 2 in two patients and a score of 3 in one patient). Two of these patients were treated with HSRT and the other patient was treated with SRS. All patients who had facial weakness were treated with a short course of oral dexamethasone. Finally only one patient still had permanent facial neuropathy (House- Brackmann score 2) at the last follow-up.

### Noncranial nerve complication

One patient developed hydrocephalus at 11 months after HSRT. Reoperation was performed at that time. This was the only patient who had an operation after HSRT.

## DISCUSSION

Management of VS requires multidisciplinary screening to select patients who are suitable for different approaches to observation, surgery, SRS/SRT, or combinations of treatment. Observation may be appropriate in selecting NF-2 and some elderly patients, but early intervention appears to be the best strategy for long-term hearing preservation in most patients [12]. Surgery appears to be the best initial treatment in patients with tumors sufficiently large enough to cause symptomatic brainstem compression with obstructive hydrocephalus. SRS and SRT should be considered the best management strategy for the majority of small to medium sized tumor VS patients [13].

Early radiosurgery series of SRS used to treat VS with higher dose single fraction (14–18 Gy) had higher rates of cranial nerve neuropathies (15–20% trigeminal and/or facial nerves, and 67% with decreased hearing function) [14]. This led to the practice of lowering the doses of the SRS (12–13 Gy) and the SRT technique. Both approaches have shown improved results (the local control was 92–100%, hearing preservation was 58–65%, trigeminal and facial nerve neuropathy was 1–5%) [7–10, 15–18]. Until now the standard stereotactic radiation fractionation for treatment of VS was still unknown due to a lack of published Level I evidence regarding this controversial issue. Because most previous non-randomized studies [7–10] compared only single versus multiple fractionation (SRS vs HSRT, or SRS vs CSRT), the aim of this study was to compare the three commonly used stereotactic radiation schedules, i.e. SRS, HSRT and CSRT, in treating VS.

Due to a major concern about hearing outcomes, the selection criteria for each treatment included both pretreatment hearing function and tumor size. SRS was only selected to treat patients who had non-serviceable hearing and small tumors (≤ 3cm); HSRT or CSRT was given to the remaining patients who were not suitable for SRS. By following this protocol, we achieved excellent local control and hearing preservation with a low complication rate. Apart from this study, there have been four reports [7–10] attempting to compare the outcomes of SRS and SRT. The first study by Andrews *et al.* [10] in 2001 reported that tumor control rates for SRS (*n* = 63) and CSRT (*n* = 46) were 98% and 97% for VS patients, respectively. In 2003, the second paper by Meijer *et al.,* [7] reported a series of 129 patients with VS treated with Linac-based SRS vs HSRT. They also found a comparable local control rate for SRS and HSRT (100% vs 94%). In 2010, Combs *et al.* [8] reported the tumor control rate was 96% for both the SRS and CSRT groups, and this was similar to the 97.9% tumor control rate for CSRT and 98.5% forSRS reported by Kopp *et al.* [9] These tumor control rates compare well with the tumor control rates of 95% for RS, 100% for HSRT, and 95% for CSRT, as found in our study.

While the fractionation schedule seems to have no impact on local control, the impact on hearing preservation rate of using different fractionation schedules is still controversial. The data from single institution reports on the effects of 12–13 Gy SRS on hearing preservation rates varies from 32–71% [15], while the best results for hearing preservation rates have been 63–94% from CSRT with a total dose of 40–57.6 Gy [19]. The third alternative, HSRT, using various fractionation schedules such as 4–5 Gy × 5, 5–6 Gy × 5, or 3 Gy × 10, showed a hearing preservation rate of 61–100% [7, 17]. The relationship between the fractionation schedule and the hearing preservation rate is somewhat conflicting in the non-randomized comparative studies. For the example, Kopp *et al.* [9] reported an 85% hearing preservation rate after SRS and 79% after CSRT, which was similar to the 78% hearing preservation rate for both SRS and SRT reported by Combs *et al*. [8]. Meijer *et al.* [7] also reported the 5-year hearing preservation probability for SRS and HSRT as 75% and 61%, respectively, but without any statistically significant difference. Nevertheless, a contradictory result was reported by Andrew *et al.* [10], whose report was 2.5 times higher for hearing preservation rate in patients who received CSRT (81%) than those who received SRS (33%), *P* = 0.0228). Table 4 shows a comparison of our study with the previous studies comparing SRS and SRT.

**Table 4.** Published studies on SRS/SRT for vestibular schwannoma

Study	Treatment/number of pt	5 year LC rate (%)	5-year hearing preservation rate (%)	5-year facial nerve preservation rate (%)	5-year trigeminal nerve preservation rate (%)
Andrew *et al.*, 2004 [10]	SRS/69 CSRT/56	98 97	33 81	98 98	95 93
Meijer *et al.*, 2003 [7]	SRS/49 HSRT/80	100 94	75 61	93 97	92 98
Comb *et al.*, 2010 [8]	SRS/30 CSRT/175	96 96	70 78	83 98	93 97
Kopp *et al.*, 2010 [9]	SRS/68 CSRT/47	97.9 98.5	79 85	100 100	87 100
Our study	SRS/39 HSRT/79 CSRT/28	95 100 95	75 87 63	98 97 100	100 99 100

SRS = stereotactic radiosurgery, HSRT = stereotactic radiotherapy, hypofraction, CSRT = stereotactic radiotherapy, conventional fraction, LC = local control.

Based on radiobiology principles, late-responding tissue such as cranial nerve and brain tissue might be subject to more injury when dose fractionation exceeding a conventional 1.8–2 Gy dose per fraction is applied. From this knowledge, we hypothesized that CSRT should have a higher hearing preservation rate than HSRT. Nevertheless, our hypothesis was not correct; CSRT did not show the improved hearing preservation we expected. However, this finding probably results from the larger tumor volumes and relatively small number of patients in the CSRT group.

The presence of NF-2 was the only factor that was associated with poorer hearing preservation, but this might have been due to the small sample size. Overall, the results obtained for NF-2 VS were not as good as those achieved in treating sporadic unilateral [20–23]. Further studies are needed to evaluate the techniques for improvement in local control and hearing preservation, especially in NF-2 patients. The pre-treatment Gardner–Robertson score may be important for hearing preservation rates. Andrews *et al.* [10] found a significantly greater probability of hearing preservation in patients with pre-treatment GR I grading, suggesting that early intervention without observation may be a favorable policy. Our results showed that the patients who had pre-treatment GR II deteriorated to GR IV after treatment more often than did GR I patients. We concluded that immediate treatment of GR I patients with SRT might yield the highest probability of functional hearing preservation.

Our study used various doses and fractionations such as 12 Gy × 1, 6 Gy × 3, 5 Gy × 4–5, 3 Gy × 10, and 2 Gy × 25. These schedules are considered to have different radiobiological effects, which may make the analysis of this study difficult. Currently, there is no reliable way to use the linear-quadratic (LQ) formula to extrapolate equivalent effects of high-dose single fractions to a fractionated course of RT for VS. The LQ model may not adequately explain dose–response relationships for either tumor or normal tissue when stretched to include the high radiation doses used with SRS. However, we hope that reliable radiobiological parameters extracted from dose–response data will be available in the near future.

The treatment technique selected for each patient in this study was based mainly on hearing function and tumor size. SRS was mostly reserved for patients with smaller sized tumors (<3 cm in maximal diameter) and non-serviceable hearing, while SRT was reserved for those with serviceable hearing. One of the reasons for this is that SRS was previously reported as one of the potential risk factors for cranial nerve injury in cases of relatively large tumor size [2, 24–25]. Although this finding has not been consistently reported in the literature [5, 26], we believe that it was prudent for us to take this issue into account before planning the fractionation schedule. Our strategy seems to have been suitable because as it turned out there was no significant difference in tumor control or adverse effect observed between the three methods. However, our selection criteria were slightly different for the two techniques as well as from other studies. Therefore, it is difficult to conclude that SRS is not as good as SRT for medium or large sized VS tumors.

## CONCLUSION

In summary, this study showed excellent outcomes for 139 VS patients treated with either SRS or SRT, without significant difference with respect to local control, hearing preservation, or complication rate. HSRT may be preferable to CSRT because it has a shorter treatment duration. However, further well-designed, randomized comparative studies of the different techniques, particularly investigating hearing preservation rates, are required.
